# An immunohistochemical study of cyclin-dependent kinase 5 (CDK5) expression in non-small cell lung cancer (NSCLC) and small cell lung cancer (SCLC): a possible prognostic biomarker

**DOI:** 10.1186/s12957-016-0787-7

**Published:** 2016-02-09

**Authors:** Kanglai Wei, Zhihua Ye, Zuyun Li, Yiwu Dang, Xin Chen, Na Huang, Chongxi Bao, Tingqing Gan, Lihua Yang, Gang Chen

**Affiliations:** 1 Department of Pathology, First Affiliated Hospital of Guangxi Medical University, No.6 Shuangyong Road, Nanning, Guangxi Zhuang Autonomous Region 530021 People’s Republic of China; 2 Department of Medical Oncology, First Affiliated Hospital of Guangxi Medical University, No.6 Shuangyong Road, Nanning, Guangxi Zhuang Autonomous Region 530021 People’s Republic of China

**Keywords:** Lung neoplasms, Cyclin-dependent kinase 5, Immunohistochemistry, Tissue array analysis, Neoplasm metastasis

## Abstract

**Background:**

Cyclin-dependent kinase 5 (CDK5) is an atypical CDK which plays a vital role in several cancers via regulating migration and motility of cancer cells. However, the clinicopathological impact and function of CDK5 in lung cancer remain poorly understood. The present study was aimed at exploring expression and clinicopathological significance of CDK5 in lung cancer.

**Methods:**

There were 395 samples of lung tissue including 365 lung tumors (339 non-small cell lung cancers and 26 small cell lung cancers) and 30 samples of normal lung. CDK5 expression was detected by immunohistochemistry on lung tissue microarrays.

**Results:**

Over expression was detected in lung cancer compared with normal lung tissues (*P* = 0.001). Furthermore, area under curve (AUC) of receiver operating characteristic (ROC) of CDK5 was 0.685 (95 % CI 0.564~0.751, *P* = 0.004). In lung cancer, we also discovered close correlations between CDK5 and pathological grading (*r* = 0.310, *P* < 0.001), TNM stage (*r* = 0.155, *P* = 0.003), and lymph node metastasis (*r* = 0.279, *P* < 0.001) by using Spearman analysis. In two subgroups of non-small cell lung cancer (NSCLC) and small cell lung cancer (SCLC), the expression of CDK5 was also higher than that of normal lung tissue, respectively (*P* = 0.001 and *P* = 0.004). Moreover, in NSCLCs, Spearman analysis revealed that expression of CDK5 was correlated with TNM stages (*r* = 0.129, *P* = 0.017), lymph node metastasis (*r* = 0.365, *P* < 0.001), and pathological grading (*r* = 0.307, *P* < 0.001), respectively. The significant correlation was also found between CDK5 expression and TNM stages (*r* = 0.415, *P* = 0.049) and lymphatic metastasis (*r* = 0.469, *P* = 0.024) in SCLCs.

**Conclusions:**

The results of this present study suggest that the CDK5 expression is associated with several clinicopathological factors linked with poorer prognosis.

## Background

Lung cancer is the most common type of cancer and the leading cause of cancer-related deaths in the world [1, 2]. In China, the incidence and the mortality of lung cancer increase rapidly, and now, lung cancer is the first dominating cancer [3]. Non-small cell lung cancer (NSCLC) is the most frequent (approximately 85 %) class of lung cancers [4, 5]. As a result of the insufficiency of efficacious biomarkers for early diagnosis, the majority of lung cancer patients are diagnosed in an advanced stage [6]. Although there are increasing proofs of therapeutic targets like EGFR, HER2, ALK, ROS1, BRAF, MET, VEGF, and FGFR1 and perpetual endeavor in clinic, the prognosis for patients with NSCLC still remains poor, with only a 5-year survival rate of 15 % with the normal therapy [7–9]. Therefore, there is an urgent requirement to discover new stable and independent biomarkers for prognosis and molecular therapy for lung cancer.

Cyclin-dependent kinases (CDKs) are serine/threonine kinases activated by cyclins [10]. CDK5 is a member of CDKs and the investigation of CDK5 in cancer is increasing. In addition to western blot analysis, immunohistochemistry has also been performed to detect expression of CDK5 in cancer tissue [11]. CDK5 has been reported to be upregulated in prostate cancer, breast cancer, medullary thyroid carcinoma, pituitary adenoma, and hepatocellular carcinoma, and CDK5 gene amplification was found in lung cancer [11–16]. However, decreased expression of CDK5 was detected in gastric cancer [17]. The results of the studies showed that CDK5 was greatly related to proliferation, migration, and motility of cancer cells [13–17]. Moreover, downregulation of CDK5 indicated higher overall survival in multiple myeloma [18]. With regard to prognostic implications, decreased expression of CDK5 was associated with advanced clinical stage and poor survival in gastric cancer patients and increased CDK5 expression was correlated to high pathological grading in breast cancer [11, 17]. So far, several articles have studied the potential role of CDK5 in lung cancer in vitro [19–22]. However, only one paper mentioned the clinical contribution of CDK5 in lung cancer with only 95 NSCLC patients and without small cell lung cancer (SCLC) cases by Liu et al. [23]. In the current study, we set up a larger sample size of 365 lung cancers, 3.8 times bigger than the previous study performed by Liu et al. [23]. Hence, the objective of this study was to explore the expression and clinicopathological significance of CDK5 in lung cancers and investigate its potential role of CDK5 as a biomarker for diagnosis and prognosis prediction for lung cancer patients.

## Methods

### Tissue samples

This study was conducted with 395 samples including 365 lung cancers and 30 normal lung tissues. The fixation was performed shorter than 15 min after surgical removal of the tissue with neutral-buffered formalin (10 %), and fixation time was 24–48 h according to the tissue size. Two pathologists (Kanglai Wei and Gang Chen) screened all the collected hematoxylin- and eosin-stained sections and selected areas of the paraffin-embedded tissue specimens that contained representative tumor or non-tumorous cells. Two tissue cores of 0.6 mm in diameter were taken from each donor block sample and arrayed into a new blank recipient paraffin block (35 mm × 22 mm × 5 mm) with a commercially available microarray instrument (Beecher Instruments, USA). Two TMA blocks included 150 cases (300 tissue cores) and the third one comprised of 95 cases (190 tissue cores), respectively. The total lung material was mounted into three blocks. The age range of lung cancer patients was from 19 to 84 years and was from 19 to 73 years of normal lung tissue. The mean age was 57.67 and 54.03 years for cancer and normal controls, respectively. When lung cancer was separated into two subgroups, there were 339 samples of NSCLCs and 26 samples of SCLCs. Furthermore, NSCLCs were composed of 127 samples of adenocarcinomas, which included four subtypes of 83 cases of acinar adenocarcinoma, 19 cases of papillary adenocarcinomas,18 cases of bronchioloalveolar cell carcinomas, and 7 cases of mucinous carcinomas. NSCLCs also included 175 squamous cell carcinomas, 28 adenosquamous carcinomas, 8 undifferentiated carcinomas, and 1 large cell carcinoma (Table 1). Various clinicopathological factors of patients were collected, including gender, age, pathological grading, TNM stage, lymph node metastasis, tumor size, and distal metastasis. The samples were obtained by random selection of the lung cancer patients by surgery without cancer-related treatment in the First Affiliated Hospital of Guangxi Medical University from January 2010 to December 2012. Approval of this study was achieved from the Ethical Committee of the First Affiliated Hospital of Guangxi Medical University, clinical doctors and patients. Moreover, two pathologists were responsible for the diagnosis, independently.

**Table 1:** The classification of lung cancer

	Cancer subtype of histology		
Lung cancer	SCLC		
	NSCLC	Squamous cell carcinomas	
		Adenosquamous carcinomas	
		Large cell carcinoma	
		Undifferentiated carcinomas	
		Adenocarcinoma	Acinar adenocarcinoma
			Papillary adenocarcinomas
			Bronchioloalveolar cell carcinomas
			Mucinous carcinomas

This table was to classify the subtypes of lung cancer. Lung cancer is consisted of non-small cell lung cancer (NSCLC) and small cell lung cancer (SCLC). NSCLC was composed of squamous cell carcinomas, adenosquamous carcinomas, large cell carcinoma, undifferentiated carcinomas, and adenocarcinoma. Furthermore, adenocarcinoma was composed of acinar adenocarcinoma, papillary adenocarcinomas, bronchioloalveolar cell carcinomas, and mucinous carcinomas

### Immunohistochemistry

Santa Cruz Biotechnology (Heidelberg, Germany) provided the CDK5 antibody (C-8,sc-173,1:50 dilution) for immunostaining, and a ZSGB Kit (PV-6000, ZSGB, Beijing, China) was used for the secondary antibody at room temperature. CDK5 immunostaining score was determined by both positive rate of stained tumor cells and staining intensity. Concretely, the positive rate of stained tumor cells and the corresponding score were assigned as follows: 0 (0 %), 1 (1–25 %), 2 (26–50 %), 3 (51–75 %), and 4 (76–100 %). The intensity of CDK5 staining was scored from 0 to 3, and the detailed standard was as follows: 0 (no staining), 1 (weak staining), 2 (moderate staining), and 3 (strong staining). Samples were scored by the summation of the percentage of CDK5-positive cells and staining intensity. The total score of immunostaining was more than two which was considered as positive expression of CDK5. Immunostaining was assessed and graded independently by two pathologists (Kanglai Wei and Gang Chen).

### Statistical analysis

The statistical analysis was conducted by SPSS 20.0 completely, and *P* values less than 0.05 were considered statistically significant. The chi-square test was used in the analysis of contrast of two groups, and when it exceeded two groups, Kruskal-Wallis H test was performed. Further, Spearman analysis was performed to study the relationship between CDK5 expression and clinicopathological characteristics. Moreover, we conducted ROC curve to evaluate the diagnostic significance of CDK5 in lung cancer, and the area under curve (AUC) of CDK5 more than 0.5 was considered significant.

## Results

### (CDK5) expression in lung cancer

**Fig. 1:**
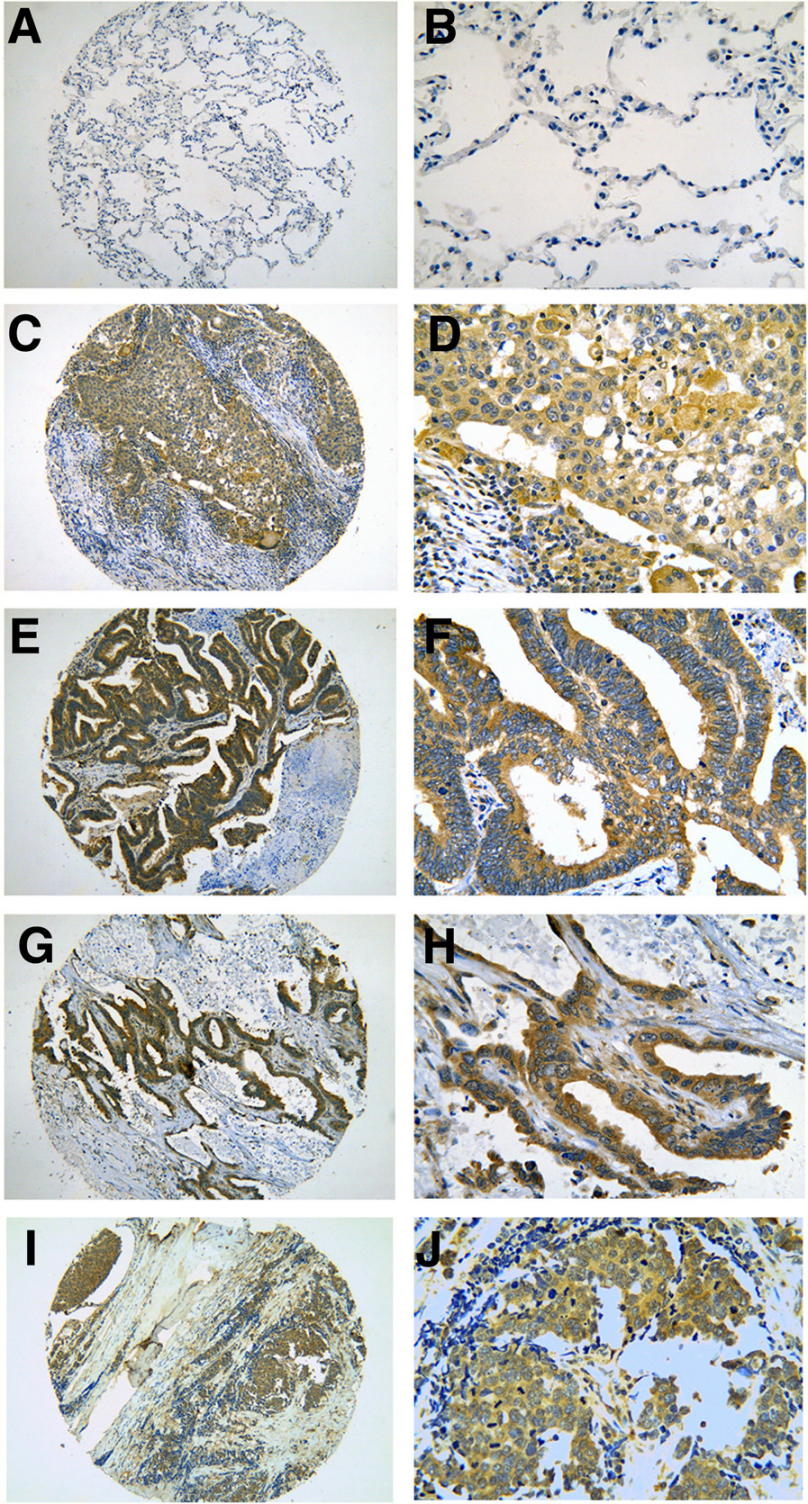
Immunohistochemical staining of CDK5 in lung tissue. Negative expression of CDK5 was found in normal lung cancer tissue (**a** ×100, **b** ×400) and significantly positive expression of CDK5 was detected in the cytoplasm of squamous carcinoma (**c** ×100, **d** ×400), papillary adenocarcinoma (**e** ×100, **f** ×400), bronchioloalveolar cell carcinoma (**g** ×100, **h** ×400), small cell lung cancer (SCLC, **i** ×100, **j** ×400)

CDK5-positive signaling located in the cytoplasm of the tumor cells. Significantly increased expression of CDK5 in lung cancer tissues (51.5 %, 188/365) was found as compared to that in normal lung tissues (20 %, 6/30, *P* = 0.001, Table 2, Fig. 1). Furthermore, we conducted ROC curve to evaluate the diagnostic significance of CDK5 in lung cancer. The AUC of CDK5 was 0.685 (95 % CI 0.564~0.751, *P* = 0.004). However, there was no statistical significance between expression of CDK5 in NSCLC and SCLC. Concerning the correlation between CDK5 expression and clinical features, CDK5 was found to be related to several clinicopathological parameters (Table 3). The positive rate of CDK5 expression was higher (68.3 %, 43/63) in advanced stages (III and IV) than in early stages (I and II) (47.8 %, 143/299, *P* = 0.003, Table 3). Higher expression of CDK5 was found in lung cancer patients with lymphatic metastasis (76.6 %, 98/128) compared to those without lymphatic metastasis (37.6 %, 88/234, *P* < 0.001, Table 3). In pathological grading III (66.4 %, 87/131), CDK5 expression was higher than that in pathological grading I (25.6 %, 10/39, *P* < 0.001) and there was an increasing trend for CDK5 positive rate as the pathological grading increased (*P* < 0.001, Table 3). In addition, Spearman coefficient of correlation was performed to investigate the relationship between the expression of CDK5 and clinicopathological parameters. It was showed that there were close correlations between CDK5 expression and TNM stage (*r* = 0.155, *P* = 0.003), lymph node metastasis (*r* = 0.279, *P* < 0.001), and pathological grading (*r* = 0.310, *P* < 0.001). A marginal correlation between CDK5 expression and distal metastasis has been found (*r* = 0.102, *P* = 0.053). Besides, no significant correlation between CDK5 expression and other clinicopathological factors was discovered, such as gender, age, and tumor diameter.

**Table 2:** CDK5 expression in lung cancer compared with normal lung tissue

Cancer and normal lung tissue			*n*	CDK5 negative (*n*, %)	CDK5 positive (*n*, %)	Z	*P*
Normal lung tissue			30	24(80.0)	6(20.0)		
Cancer tissue			365	177(48.5)	188(51.5)	−3.314	0.001
SCLC			26	11(42.3)	15(57.7)	−2.880	0.004
NSCLC			339	166(49.0)	173(51.0)	−3.225	0.001
	Squamous cell carcinoma		175	88(50.3)	87(49.7)	−3.013	0.003
	Adenosquamous carcinoma		28	10(35.7)	18(64.3)	−3.392	0.001
	Undifferentiated carcinoma		8	3(37.5)	5(62.5)	−2.324	0.020
	Large cell carcinoma		1	1(100)	0(0)	−0.490	0.624
	Adenocarcinoma		127	64(50.4)	63(49.6)	−2.929	0.003
		Acinar adenocarcinoma	83	36(43.4)	47(56.6)	−3.430	0.001
		Papillary adenocarcinoma	19	12(63.2)	7(36.8)	−1.288	0.198
		Bronchioloalveolar cell carcinoma	18	10(55.6)	8(44.4)	−1.785	0.074
		Mucinous carcinoma	7	6(85.7)	1(14.3)	−0.343	0.732

Non-small cell lung cancer (NSCLC) vs small cell lung cancer (SCLC) *P* = 0.513

**Table 3:** CDK5 expression associated with the various clinicopathological parameters in lung cancer

Lung cancer	*n*	CDK5 negative (*n*, %)	CDK5 positive (*n*, %)	*Z*	*P*
Gender				−0.884	0.377
Male	275	137(49.8)	138(50.2)		
Female	90	40(44.4)	50(55.6)		
Age(years)				−0.418	0.676
<60	196	96(49.0)	100(51.0)		
≥60	169	81(47.9)	88(52.1)		
Pathological grading				25.060^a^	<0.001
I	39	29(74.4)	10(25.6)		
II	92	53(57.6)	39(42.4)		
III	131	44(33.6)	87(66.4)		
TNM				−2.944	0.003
I–II	299	156(52.2)	143(47.8)		
III–IV	63	20(31.7)	43(68.3)		
LNM				−7.080	<0.001
Yes	128	30(23.4)	98(76.6)		
No	234	146 (62.4)	88(37.6)		
Tumor diameter (cm)				−1.653	0.098
≤7	314	158(50.3)	156(49.7)		
>7	48	18(37.5)	30(62.5)		
Distal metastasis				−1.931	0.054
Absent	346	172(49.7)	174(50.3)		
Present	16	4(25.0)	12(75.0)		

^a^Kruskal-Wallis *H* test was performed between the groups of pathological grading

### Cyclin-dependent kinases (CDK5) expression in non-small cell lung cancer (NSCLC)

When lung cancer patients were separated into two subgroups of NSCLC and SCLC, we discovered that there was higher positive rate in NSCLC compared with normal lung tissues (*P* = 0.001, Table 4). Higher CDK5-positive expression was also found in the subgroups of NSCLCs including adenocarcinoma (*P* = 0.003), squamous cell carcinoma (*P* = 0.003), adenosquamous carcinoma (*P* = 0.001), and undifferentiated carcinoma (*P* = 0.02), than that in the normal lung tissue. After adenocarcinoma was further split into four different types, remarkably higher expression of CDK5 was found in acinar adenocarcinoma as compared to normal lung tissues (*P* = 0.001, Table 4). When the relationship between CDK 5 expression and other parameters was concerned in the patients with NSCLCs, higher CDK5 expression positive rate appeared in the advanced stages (III and IV) (66 %, 35/53) compared with the early stages (I and II) (48.3 %, 138/286, *P* = 0.018, Table 4) and the similar result was found in lymph node metastasis (76.5 %, 88/115) as compared to non-lymph node metastasis (37.9 %, 85/224, *P* < 0.001, Table 4). In pathological grading III, the positive expression of CDK5 was 66.2 % (86/130) in the cases of NSCLC higher than that in pathological grading I (25.6 %, 10/39)and II (42.4 %, 39/92, both *P* < 0.001, Table 4). Borderline difference of CDK5 expression has been found between distal metastasis (75 %, 12/16) and non-distal metastasis (49.8 %, 161/323, *P* = 0.05, Table 4). Moreover, Spearman analysis showed that the positive CDK5 expression in NSCLC was correlated with TNM stages (*r* = 0.129, *P* = 0.017), lymph node metastasis (*r* = 0.365, *P* < 0.001), and pathological grading (*r* = 0.307, *P* < 0.001). A marginal correlation between CDK5 expression and distal metastasis was also noticed (*r* = 0.107, *P* = 0.05).

**Table 4:** The correlation of CDK5 with diverse clinical clinicopathological factors in NSCLC

NSCLC	*n*	CDK5 negative (*n*, %)	CDK5 positive (*n*, %)	Z	*P*
Gender				−0.406	0.685
Male	254	126(49.6)	128(50.4)		
Female	85	40(47.1)	45(52.9)		
Age(years)				−0.080	0.936
<60	181	89(49.2)	92(50.8)		
≥60	158	77(48.7)	81(51.3)		
Pathological grading				24.58^a^	<0.001
I	39	29(74.4)	10(25.6)		
II	92	53(57.6)	39(42.4)		
III	130	44(33.8)	86(66.2)		
TNM				−2.376	0.018
I–II	286	148(51.7)	138(48.3)		
III–IV	53	18(34.0)	35(66.0)		
LNM				−6.717	<0.001
Yes	115	27(23.5))	88(76.5)		
No	224	139(62.1)	85(37.9)		
Tumor diameter (cm)				−1.145	0.252
≤7	295	148(50.2)	147(49.8)		
>7	44	18(40.9)	26(59.1)		
Distal metastasis				−1.962	0.05
Absent	323	162(50.2)	161(49.8)		
Present	16	4(25.0)	12(75.0)		
Histology				3.646^a^	0.456
Adenocarcinoma	127	64(50.4)	63(49.6)		
Squamous cell carcinoma	175	88(50.3)	87(49.7)		
Adenosquamous carcinoma	28	10(35.7)	18(64.3)		
Undifferentiated carcinoma	8	3(37.5)	5(62.5)		
Large cell carcinoma	1	1(100)	0(0)		
Adenocarcinoma classification				6.508^a^	0.089
Acinar adenocarcinoma	83	36(43.4)	47(56.6)		
Papillary adenocarcinoma	19	12(63.2)	7(36.8)		
Broncholoalveolar cell carcinoma	18	10(55.6)	8(44.4)		
Mucinous carcinoma	7	6(85.7)	1(14.3)		

Pathological grading I vs. II *Z* = −1.805, *P* = 0.071, I vs. III *Z* = −4.466, *P* < 0.001, II vs. III *Z* = −3.508, *P* < 0.001. Acinar adenocarcinoma vs. mucinous *Z* = −2.144, *P* = 0.032. There were no differences of expression of CDK5 in other subgroups

^a^Kruskal-Wallis H test was performed when the data were divided into more than two groups

### Cyclin-dependent kinases (CDK5) expression in small cell lung cancer (SCLC)

There were 26 patients of SCLC, and the positive rate of CDK5 expression was 57.7 % (15/26), significant higher compared to normal lung tissues (20 %, 6/30, *P* = 0.004). In the patients with SCLC, higher expression of CDK5 was found in female (100 %, 5/5) and lymph node metastasis (76.9 %, 10/13) compared with that in male (47.6 %, 10/21, *P* = 0.037) and without lymph node metastasis (30 %, 3/10, *P* = 0.028, Table 5), respectively. Spearman coefficient of correlation showed that the positive CDK5 expression in SCLC was correlated with gender (*r* = 0.418, *P* = 0.034), TNM stages (*r* = 0.415, *P* = 0.049), and lymph node metastasis (*r* = 0.469, *P* = 0.024, Table 5).

**Table 5:** The correlation of CDK5 expression with various clinical pathological factors in SCLC

SCLC	*n*	CDK5 negative (*n*, %)	CDK5 positive (*n*, %)	*Z*	*P*
Gender				−2.089	0.037
Male	21	11(52.4)	10(47.6 )		
Female	5	0(0)	5(100)		
Age(years)				−0.515	0.606
<60	15	7(46.7)	8(53.3)		
≥60	11	4(36.4)	7(63.6)		
TNM				−1.948	0.051
I–II	13	8(61.5)	5(38.5)		
III–IV	10	2(20.0)	8(80.0)		
LNM				−2.201	0.028
Yes	13	3(23.1)	10(76.9)		
No	10	7(70.0)	3(30.0)		
Tumor diameter (cm)				−1.888	0.059
≤7	19	10(52.6)	9(47.4)		
>7		0(0)	4(100)		

## Discussion

Cyclin-dependent kinase 5 (CDK5) is vital in neural cell migration and differentiation and is activated by p35 or p39 [24], and CDK5 is considered to be essential in neuronal cells [25, 26]. Nevertheless, as a unique member of cyclin-dependent kinases, the function of CDK5 beyond the nervous system has been demonstrated. CDK5 also regulates cell proliferation by alterant expression and its downstream signaling pathways, especially in cancer cells. Up to date, there has been a growing number of evidence that CDK5 has an important effect on cancer progression [27]. The expression of CDK5 was aberrant in several cancers, and CDK5 regulated the proliferation of cancer cell in prostate cancer, medullary thyroid carcinoma, and gastric cancer [14, 15, 17]. In breast cancer, CDK5 was essential for the motility of cancer cell [11]. Moreover, CDK5 can be used to predict the prognosis of multiple myeloma [18]. In a word, the role of CDK5 in cancer is attracting increasing attention. To date, the expression of CDK5 was investigated in several cancers [13, 15, 17]. Higher expression of CDK5 was observed in hepatocellular carcinoma, ampullary adenocarcinoma, breast cancer, and medullary thyroid carcinoma, and Zachary et al. confirmed the expression of CDK5 was upregulated in colorectal, head/neck, breast, lung, ovarian, lymphoma, prostatic, sarcoma, myeloma, and bladder cancers via the Oncomine microarray online data mining software [11, 12, 18, 28]. However, downregulated expression of CDK5 was observed in gastric cancer. Thus, CDK5 might be heterogeneously expressed in different cancers.

In the present study, immunohistochemistry on lung tissue microarrays was performed to explore the expression of CDK5 in lung cancer and normal lung tissues. There was prominently higher expression of CDK5 in lung cancer, independent of various pathological subtypes, than in normal lung tissue. In the study of Liu et al., CDK5 was upregulated in cancer tissue as compared to benign pulmonary disease with a sample size of 95 non-small cell lung cancers (NSCLCs) [23]. Our study, with a bigger sample size, approximately four times, confirmed that increased expression of CDK5 could be detected in lung cancer tissue compared with normal lung tissue and further supported that CDK5 was considered as an oncogene in lung cancer. No significant difference of CDK5 expression was found between NSCLC and SCLC in this current study. Although NSCLC and SCLC are commonly regarded as different diseases owing to their distinct biology and genomic abnormalities, the role and function of CDK5 may be consistent, as CDK5 level was both upregulated in NSCLC and SCLC tissues than the non-cancerous lung. However, the exact role of CDK5 in SCLC needs further investigation, since only a limited sample size (*n* = 26) was included in the current study. Taken together, CDK5 might be a potential biomarker of lung cancer despite its histology types.

The regulative mechanism of CDK5 in several cancers was investigated. CDK5 regulates DNA damage response via phosphorylating Ataxia telangiectasia mutated (ATM) kinase and thereby affecting its downstream signal pathways which was crucial to progression of hepatocellular carcinoma [12]. In ampullary adenocarcinoma, over expression of nestin/CDK5 was involved in several oncogenic pathways (the activation of NOTCH, TGF-β1, or PDGFR pathways) that facilitated invasiveness of cancer [28]. In breast cancer, CDK5 takes part in epithelial-mesenchymal transition induced by TGF-β1which is vital for tumor metastasis [11]. In medullary thyroid carcinoma, CDK5 is essential to tumorigenesis and progression by retinoblastoma protein (Rb) and inhibition of Rb reduced proliferation of medullary thyroid carcinoma [15]. The mechanisms of tumorigenesis and progression in lung cancer might be similar to the mechanisms aforementioned in other cancers in consideration of a consistent trend of CDK5 expression. However, this hypothesis needs to be verified with in vitro and in vivo studies.

Though expression of CDK5 was detected in lung cancer tissue and regulative mechanism of CDK5 was investigated in other cancers, the mechanism and exact role of CDK5 in the carcinogenesis and development of lung cancer remain unclear. A study of Korean population shows that CDK5 promoter polymorphisms contribute to the genetic susceptibility to lung cancer [20]. As one of the downstream components of the EGFR-family-signaling pathway, the gene of CDK5 was amplified in lung cancer and it might be the common mechanism of oncogene activation in carcinogenesis [16]. Tripathi et al. demonstrated that the CDK5 phosphorylates four serines located N-terminal to the Rho-GTPase activating protein(Rho-GAP) domain in DLC1(deleted in lung cancer 1), a tumor suppressor protein, and thereby activates DLC1 [22]. Through the reconstruction of an integrated genome-scale co-expression network, Bidkhori et al. exhibited that CDK5 played a vital role in cell cycle progression in lung adenocarcinoma [19]. The studies above of CDK5 in lung cancer suggested CDK5 may play an oncogenic role in lung cancer.

In addition to the expression of CDK5 in cancer tissues, the relationship between CDK5 and pathological parameters has been paid more and more attention to. There were a few researches of the relationship between CDK5 and clinical factors in the patients with cancers. In breast cancer, upregulated expression of CDK5 was related to higher grading (grading III) [11]. In multiple myeloma, downregulated expression of cdk5 predicted favorable overall survival after bortezomib treatment [18]. Liu et al. demonstrated that higher expression of CDK5 was correlated with low/undifferentiated, high pathological stage, lymph node metastasis, shorter median survival, and lower 5-year overall survival in the patients with NSCLC [23]. Similarly, with a larger sample size, the consistent trend in this current study was confirmed that higher positive rate of CDK5 expression was greatly correlated with unfavorable clinicopathological parameters, including advanced TNM stage, lymphatic metastasis, and high pathological grading, which commonly indicate poorer prognosis. Thus, CDK5 might be used for the prediction to prognosis of lung cancer.

Further, Demelash et al. [21] demonstrated that CDK5 played a vital role in the regulation of lung cancer cell migration and invasion through Wound closure and Boyden chamber assay and certified that achaete-scute homologue-1 (ASH1), a basic transcription factor which was expressed in lung cancer cells with neuroendocrine features [29], could stimulate migration of lung cancer cells through CDK5/p35 pathway. The mechanism may support that CDK5 was closed related to lymphatic metastasis in lung cancer.

## Conclusions

In summary, in this current study, the expression of CDK5 was investigated by lung tissue microarrays and immunohistochemistry. We demonstrated that CDK5 was highly expressed in lung cancer, including non-small cell lung cancer and small cell lung cancer, compared to normal lung tissue. Higher positive rate of CDK5 was associated with several clinicopathological parameters, which are representative of the progression and deterioration of lung cancer. These results suggest that the CDK5 expression associated with several unfavorable clinicopathological factors linked with poorer prognosis. Nevertheless, further plans are needed to explore the potential function of CDK5 in vitro and in vivo in the carcinogenesis of and progression in lung cancer.
